# Passive Sampling of Gaseous Elemental Mercury Based on a Composite TiO_2_NP/AuNP Layer

**DOI:** 10.3390/nano8100798

**Published:** 2018-10-07

**Authors:** Antonella Macagnano, Paolo Papa, Joshua Avossa, Viviana Perri, Marcello Marelli, Francesca Sprovieri, Emiliano Zampetti, Fabrizio De Cesare, Andrea Bearzotti, Nicola Pirrone

**Affiliations:** 1 Institute of Atmospheric Pollution Research—National Research Council (IIA-CNR), Research Area of Rome 1, Via Salaria km 29,300, 00016 Monterotondo, Italy; p.papa@iia.cnr.it (P.P.); joshua.avossa@iia.cnr.it (J.A.); v.perri@iia.cnr.it (V.P.); e.zampetti@iia.cnr.it (E.Z.); decesare@unitus.it (F.D.C.); a.bearzotti@iia.cnr.it (A.B.); 2 Department of Innovation in Biological Systems, Food and Forestry University of Tuscia (DIBAF), Via S. Camillo de Lellis, 00100 Viterbo, Italy; 3 Institute of Molecular Science and Technologies—National Research Council (ISTM-CNR), Via G. Fantoli 16/15, 20138 Milano, Italy; m.marelli@istm.cnr.it; 4 Institute of Atmospheric Pollution Research—National Research Council (IIA-CNR), Division of Rende, c/o UNICAL-Polifunzionale, 87036 Arcavacata di Rende (CS), Italy; sprovieri@iia.cnr.it (F.S.); pirrone@iia.cnr.it (N.P.)

**Keywords:** TiO_2_NPs, AuNPs, photocatalysis, mercury vapors adsorbing layer, PAS device

## Abstract

Passive sampling systems (PASs) are a low cost strategy to quantify Hg levels in air over both different environmental locations and time periods of few hours to weeks/months. For this reason, novel nanostructured materials have been designed and developed. They consist of an adsorbent layer made of titania nanoparticles (TiO_2_NPs, ≤25 nm diameter) finely decorated with gold nanoparticles. The TiO_2_NPs functionalization occurred for the photocatalytic properties of titania-anatase when UV-irradiated in an aqueous solution containing HAuCl_4_. The resulting nanostructured suspension was deposited by drop-casting on a thin quartz slices, dried and then incorporated into a common axial sampler to be investigated as a potential PAS device. The morphological characteristics of the sample were studied by High-Resolution Transmission Electron Microscopy, Atomic Force Microscopy, and Optical Microscopy. UV-Vis spectra showed a blue shift of the membrane when exposed to Hg^0^ vapors. The adsorbed mercury was thermally desorbed for a few minutes, and then quantified by a mercury vapor analyzer. Such a sampling system reported an efficiency of adsorption that was equal to ≈95%. Temperature and relative humidity only mildly affected the membrane performances. These structures seem to be promising candidates for mercury samplers, due to both the strong affinity of gold with Hg, and the wide adsorbing surface.

## 1. Introduction

A thin film is commonly thought of as a layer with a thickness ranging from fractions of one nanometer to several micrometers; however, it is difficult to draw a border line between thick and thin films on the basis of their thickness, overall when the film is a nanocomposite or hybrid structure [1,2]. When a layer is designed to selectively adsorb gas or VOCs (volatile organic compounds), it has to be both chemically (to favor more specific interaction forces between adsorbent and adsorbate) and physically (to increase the number of the adsorbing sites, e.g., by acting on roughness and porosity) treated. The surface layer in contact with the environment is the main responsibility of the adsorbing process, and the relationship between the gas phase concentration and the adsorbed phase concentration at a constant temperature is reported as an adsorption isotherm, whereas the shape depicts the affinity relationships between the adsorbate and the absorbent [3]. Therefore, most of the chemical sensors and samplers for monitoring mercury in the atmosphere have been designed with these features taken into account. Mercury is a toxic pollutant, and it is considered by WHO (World Health Organization) as one of the top 10 chemicals of major public health concern [4]. It is continuously traveling through water, soil, and atmosphere, in various forms, to different parts of the world, and it is commonly emitted from both natural sources, as volcanoes, wildfire, and soil, and human activities, as fossil fuel burning, waste incineration, power plants, and artisanal mining [5,6]. There is a huge and quickly growing body of scientific literature on the distribution of mercury in several ecosystems: atmosphere is the principal transport pathway of Hg emissions, whereas soil and water play a significant role in the reallocation of Hg in several ecosystems [7]. Mercury in the atmosphere can be carried as gaseous elemental mercury (GEM) and gaseous oxidized mercury (GOM), which together are named as total gaseous mercury (TGM, C_mean_: ≈ 1.4 ± 0.15 ng m^−3^) and particle-bound mercury (PBM). Among them, GEM holds the most long atmospheric residence time (≈1 year) due to the relatively high vapor pressure and inertness to atmospheric oxidation [5,8]; conversely, GOM and PBM have much shorter atmospheric stays, being put down closer to their source locations [9,10]. Adsorbents based on sulfur-treated carbons, alumina, and zeolites, have been among the most commonly investigated materials that are able to capture mercury from the environment [11]. More recently, ZnS nanoparticles (NPs) have been developed and variously implemented as alternatives to remove Hg^0^ from polluted environments [12]. The strong affinity between heavy metals (e.g., mercury) and noble metals, such as gold and silver, has been also investigated in literature as a suitable strategy to capture and reveal such a pollutant in air. Therefore, a series of filters, and detecting systems exploiting such features, have been designed to remove and detect, respectively, these pollutants within the environment. Specifically, when a removal process is required, as well as the detection of a very low concentration, cartridge-like structures with a high surface-volume ratio are preferred [13,14], and large amounts of volumes containing the pollutant are fluxed throughout the cartridge/filter, entrapping it inside. Conversely, when real-time detection or diffusion processes are investigated, thin film structures are commonly preferred. Both sensors and adsorbing devices based on metal thin films, and more recently, porous or nanostructured, have been reported in literature [15,16,17]. Nanostructured thin film layers, made with nanoparticles, are preferentially assembled in ordered structures, conforming to the surface with precise control over chemical and physical properties, in reproducible scaffolding. Some examples of deposition techniques that are commonly used include the electrodeposition of metal oxide and metal nanoparticles [18], the deposition of nanoparticle monolayers via the Langmuir Blodgett technique [19], sol–gel chemistry-based deposition of nanoparticles [20], layer-by-layer dip coating [21], in situ synthesis of nanoparticles using polymeric thin films as templates [22,23] self-assembling [24], and electrospray [25]. Further, depending on the functionalization or charges on the nanoparticle (NP) shells, ordered thin layer or 3D structures can also be designed by drop-casting, which is one of the simplest and cheapest deposition techniques [26,27], even if it is rarely able to assemble homogeneous layers, especially on large surfaces, mainly due to differences in evaporation rates through the substrate, or concentration fluctuations that can lead to variations in internal structure and film thickness. When the substrate is a porous matrix, the dropped nanocomponents, depending on their affinity to the surface, can penetrate and decorate the pores’ surfaces, thus assuming a peculiar 3D-structure that is more or less conformal to the substrate scaffold. Such a porous layer, comprising interconnected void volumes and high surface-area-to-volume ratio, facilitates gas and VOC diffusion through its bulk, as well as gas/VOC adsorption onto its binding sites.

In the present study, highly pure quartz (SiO_2_) microfibrous filters have been used as suitable substrates decorated with a nanocomposite material by dropping, capable of adsorbing, and then quantifying vapors of mercury from the atmosphere, as a promising thin structure for passive air sampler (PAS). The latter are generally designed to be cheap, simple to operate, and to work without electricity. For mercury analysis, the most basic requirement is that a PAS is able to sorb a sufficient amount of mercury for accurate and precise quantification. The peculiarity of the passive samplers relies on unassisted molecular diffusion of gaseous agents (i.e., volatile vapors of elemental mercury) through a diffusive surface onto an adsorbent material. Unlike active (pumped) sampling, passive samplers require no electricity (expensive pumps), have no moving parts, and are simple to use (no pump operation or calibration). After sampling, the adsorbed mercury should be desorbed off the adsorbent by solvent (chemical procedure) or thermal desorption (physical procedure). Passive samplers have to be commonly compact, portable, unobtrusive, and inexpensive. They are able to give information on the average pollution levels over time periods of a few hours, to weeks/months. They do not require supervision, and can be used in hazardous environments. Passive samplers have been designed, using a variety of synthetic materials (like sulfur-impregnated carbon (SIC), chlorine-impregnated carbon (CIC), bromine-impregnated carbon (BIC), gold-coated sorbents (GCS), etc.) and housings for Hg collection [28,29]. Activated carbon is suggested to be the most suitable sorbent material for PASs, since it is low cost and provides a large surface area [30]. On the other hand, the amalgamation between Au and Hg is also considered as an effective alternative strategy to design mercury samplers, even if it is not very popular since more expensive [28]. However, a large variety of materials based on nanotechnology have already been applied for this purpose, even if the state of art in those nanomaterial-based passive samplers is still in the early stages [31,32,33,34,35,36,37]. Sampling rate and adsorption capacity are the two key factors to evaluate the performance of a passive sampler. The PAS sampling rate depends on the shape of the sampler, and it is affected by meteorological factors [38,39,40]; meanwhile, the adsorption capacity depends on the affinity between the adsorbate and adsorbent, as well as the adsorbing layer structure (i.e., specific surface area, pore size distribution and number of binding site), and it is affected by temperature and potential chemical interferents in the air. All of these samplers work on the basis of diffusion. Most commercially available passive/diffusive samplers are planar or axial in shape [31]. Commonly, the adsorbing matrix is disk-shaped structured, with different thicknesses and porosities. Alternatively, they can have a radial shape, consisting of a columnar sorbent surrounded by a cylindrical diffusive barrier, with the purpose of increasing the sampling rate by maximizing the surface area across which diffusion occurs (Radiello^®^ [41]). Other passive samplers for mercury vapor collection on the basis of diffusion have been constructed using a variety of synthetic materials (i.e., gold and silver surfaces, and sulfate-impregnated carbon) and housings [31,39,42].

In this work, an alternative approach adopted in the place of conventional ones has been described, designing onto a SiO_2_ micro-fibrous filter, a coating (partially penetrated inside the filter) made of an aggregation of densely packed nanoparticles of TiO_2_ finely decorated with smaller nanoparticles of gold AuNPs. Such a layer was achieved by exploiting the capability of TiO_2_ (anatase) to photoreduce aqueous HAuCl_4_ into elemental gold when irradiated with UV-light [43]. Furthermore, when a polymer (e.g., polyvinylpyrrolidone, PVP) was added to the HAuCl_4_ aqueous solution, globose gold nanoparticles could be grown onto the metal oxide particles, obtaining a nanocomposite material with promising properties in adsorbing mercury from the atmosphere. These novel nanocomposite structures have here been considered as being very attractive adsorbing layers for passive sampling, due to both the strong affinity between mercury and gold, wide adsorbing surface due to the nanosize of the materials (expected high efficiency and lifetime), the robustness of the materials and the chance to use them for many times, thus avoiding the spread of waste materials into the environment. 

## 2. Materials and Methods

All chemicals were acquired from Sigma Aldrich (Milan, Italy), and used without further purification: polyvinylpyrrolidone (PVP, *M*n = 1,300,000 g/mol), titanium (IV) oxide (anatase, ≤25 nm diameter, Sigma Aldrich, Milan, Italy) and gold (III) chloride hydrate (HAuCl_4_, 99.999%). Ultrapure water (5.5 10^−8^ S cm^−1^) was produced by MilliQ-EMD Millipore. Quartz slice filters (Whatman^TM^, Little Chalfont, UK) were 400 µm thick, and 2 cm wide with ≈2 µm pore size and ≤3 µm fiber diameter. 

Titanium (IV) oxide (anatase) were suspended in an aqueous solution of PVP/ HAuCl_4_ for preliminary investigations (600 mg TiO_2_/ 60 mL PVP_aq_/ 0.03 mg HAuCl_4_). Such a suspension was UV-irradiated for 1 hr (365 nm, Helios Italquartz, Italy), thus changing the color from yellow to blue-violet, and subsequently centrifuged and rinsed with water at least three times to remove PVP excess (Thermo Scientific SL16R 230V, Langenselbold, Germany; *T*: 4 °C; t: 15 min (×3); RCF: 5000 g). The resulting precipitated was vortexed, diluted with a few cc of ultrapure water, and deposited on the quartz slices by drop casting using a customized mask of Teflon^®^ (*d*: 11.25 mm, ≈10 mg), then heated to 80 °C, and finally to 450 °C under a clean air flow, in order to remove both possible traces of polymer and mercury absorbed during preparation. 

The nanostructured layers were analyzed by UV-Vis spectrophotometry (Spectrophotometer UV-2600, Shimadzu, integrating sphere ISR-2600Plus, Duisburg, Germany) before and after gold nano-functionalization, and by AFM (Nanosurf Flex-AFM, Liestal, Switzerland), which captured the layer surface images in tapping mode using 190Al-G tips, 190 kHz, 48 N/m. 

Powder samples for transmission electron microscopy (TEM) measurements were gently grounded in an agate mortar, dispersed in isopropyl alcohol, sonicated for 10 minutes, and dropped onto a holey-carbon coated copper TEM grid. TEM and scanning transmission electron microscopy (STEM) analysis were performed by a ZEISS Libra 200FE microscope (Oberkochen, Germany) after complete solvent evaporation overnight in air. The size distribution were manually calculated counting more than 400 NPs by iTEM software (Olympus SIS, Muenster, Germany). 

Optical micrographs were provided by Zeiss Axiophot Stereomicroscope equipped with a color videocamera (Axio Cam MRC, Wexford, Ireland) using a computer assisted image analysis system (AxioVision, Wexford, Ireland). The side-view of the quartz slice coated with AuNPs/TiO_2_NPs film was provided by a Portable USB Digital Microscope 1×–5000× Magnification Mini Microscope Camera (1x-5000X, Bangweier, Guangdong, China) by placing the sample between a microscope slide and a base support: the remaining floating sample was 45° tilted and displayed in the same picture. 

A prototype of thermal desorption system was also planned in CNR-IIA and developed (Spaziani Rolando, Italy) in order to be connected to the most commons analytical systems of mercury. The prototype was manufactured in quartz and housed in a heater system (De Marco Forneria, Italy) to allow the fast desorption of the Hg^0^ adsorbed on the thin layer of the nanostructured material, flowing pure (Air 5.0, Praxair-Rivoira, Italy) throughout the desorption chamber.

Mercury Vapor Analyzer Tekran 2537A (Tekran Instruments C., Toronto, ON Canada) was used to quantify the mercury desorbed from the nanocomposite film. The exposure of the adsorbing layers to injected volumes (µL) of mercury vapors (Tekran 2505, Mercury Calibration Unit) were carried out within customized sealed Duran glass samplers (V: 8.5 mL) to study the efficiency of the membrane. Conversely, the AuNPs-TiO_2_NPs adsorbing discs were placed into customized glass samplers with diffusive caps (nylon membrane) for sampling rate calculation, and deployed with concentrations of mercury in relative humidity (%RH)- and temperature (T)-controlled measuring rooms. Such measuring rooms were monitored by the mercury vapor analyzer and a humidity-temperature transmitter (Relative Humidity and Temperature Probe HMP230, Vaisala Corporation, Helsinki, Finland). 

## 3. Results and Discussion

**Figure 1:**
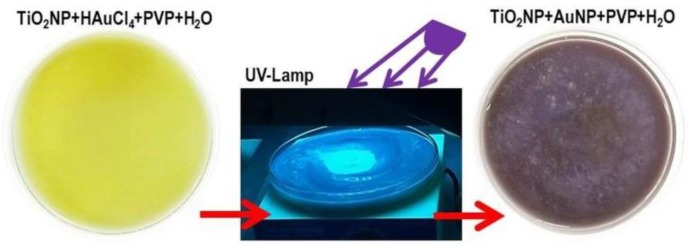
Functionalization of TiO_2_ nanoparticles by UV-irradiation in a 0.1 M PVP (polyvinylpyrrolidone) aqueous suspension.

Exploiting the photocatalytic properties of TiO_2_, gold nanoparticles were selectively grown under UV light irradiation on titania nanoparticles through the photoreduction of HAuCl_4_ in the presence of an organic capping reagent (PVP). The light yellow-colored aqueous suspension of TiO_2_NPs containing HAuCl_4_ and PVP when exposed to UV-light irradiation for 1 hr under magnetic stirring assumed a blue-purple color, due to the formation of gold nanoparticles (Figure 1). Subsequently to the centrifugation and then the resuspension of the pellet by ultrapure water, known amounts of the heterogeneous mixture, under stirring, were picked up and deposited onto several substrates to be characterized. Before each morphological and physical-chemical measurements, the samples were heated at 450 °C per 1 hr to eliminate the PVP traces and the potential mercury adsorbed.

**Figure 2:**
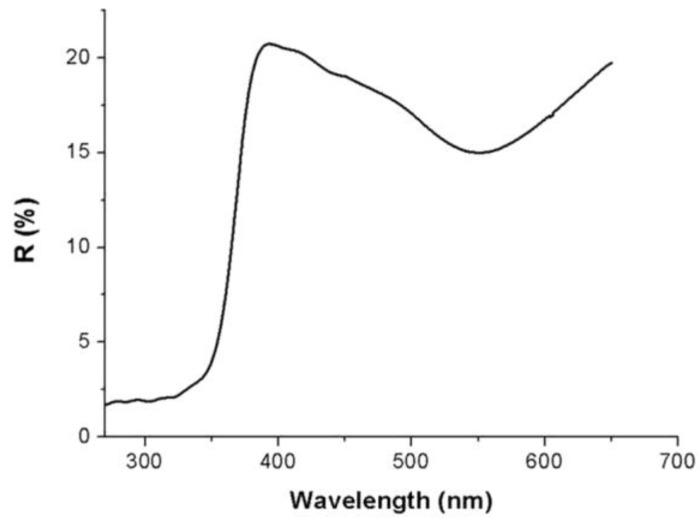
UV-Vis diffuse reflectance spectrum of AuNPs/TiO_2_ layer in 270–670 nm wavelength range.

Due to the inhomogeneity of the film, all the spectra here depicted were carried out only over a selected area of the Au/TiO_2_NPs film coating a flat substrate (SiO_2_ wafer). The sample reported a reflectance minimum (about 15%) at 550 nm wavelength, thus confirming the growth of metal gold on the TiO_2_ nanoparticles, and another stronger signal, with an onset at 380 nm, related to the charge transfer from the valence band to the conduction band of the titania nanoparticles [44]. The UV-Vis diffuse reflectance spectrum is depicted in Figure 2. The broad band (550 nm) was attributable to the characteristic localized surface plasmon resonance (LSPR) band of AuNPs, ranging between 500 and 600 nm [45,46]. This visible band was presumed to arise from the combined oscillations of the valence electrons confined in a cage of nanometer dimensions [47]: the position and shape of the surface plasmon band is affected by many parameters, including the dielectric constant of the medium, the particle size and shape and the coulomb charge of the nanoparticle. When AuNPs are joined to metal oxides, such as TiO_2_, the material appears to be purple-brown colored, due to the characteristic surface plasmon band of gold.

However, the minimum value of the reflectance band could be blue- or red-shifted, depending on the value of the average size of particles (i.e., the peak absorbance wavelength increases with particle diameter), as their aggregation (which is enhanced by a red-shift in the spectrum, as well as the broadening of adsorption peaks, and a decrease in peak intensities), functionalization, and inter-particles distance [48]. In literature, it was observed that the surface plasmon oscillation of gold nanoparticles in a suspension red shifted from ~520 to 530 nm as the particle diameter increased from 5 to 40 nm [49]. 

Both AuNP shape and size should be mainly related to the PVP concentration (the capping agent) and UV-light intensity, respectively [43,50] over the photocatalytic process. Specifically, the average particle size decreases as the intensity of the light increases. This effect of light intensity on the gold particle size could be very general, and it could be used to tune the average particle size to the optimum value when preparing Au/TiO_2_ using this route. Additionally, both the size and the number (or density) of the nanoparticles can increase directly with the duration of irradiation. 

**Figure 3:**
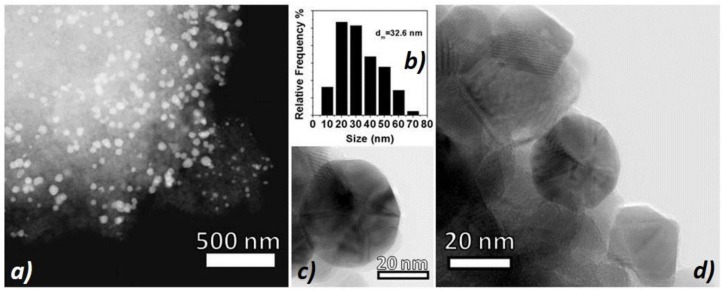
(**a**) A representative STEM micrograph of an Au/TiO_2_ anatase sample at low magnification—AuNPs (gold nanoparticles) appear brighter over the greyish support; (**b**) AuNPs size distribution and representative HRTEM (High-Resolution Transmission Electron Microscopy) micrographs of (**c**) a gold NP and (**d**) a AuNP/TiO_2_NPs cluster where a AuNP appears rounder and slightly darker than anatase ones.

The gold nanoparticle size distribution of the sample (Figure 3b) was centered around the mean value of 32.6 nm (70% of NPs ranging between 5 to 40 nm). A STEM micrograph (Figure 3a) showed a good particle dispersion onto the support (Figure 3b), whereas the HRTEM (High-Resolution Transmission Electron Microscopy) ones (Figure 3c,d) revealed well-shaped and highly crystalline gold nanoparticles that were in intimate contact with the anatase crystalline support (AuNPs appear slightly darker with respect to the anatase support). Interestingly, the gold NPs shared similar sizes with the support grains, assembling together into homogeneous aggregates.

Exploiting the properties of gold nanostructures due to the mercury adsorption, miniaturized sensing devices were demonstrated to be able to detect picograms of mercury in the air, like gold-microcantilevers [16] by changing their resonant frequency in real time. Au-TiO_2_NPs deposited onto gold electrodes have been investigated as electrochemical sensors to detect Hg (II) in water [51]. Nanocomposite sensors made of titania nanofibers decorated with gold nanoparticles showed a limit of detection of 6 pptv (parts per trillion by volume) and 2 pptv, respectively, depending on the strategy of sampling [52]. Furthermore, devices based on resistivity changes in very thin gold films are also commercially available (Jerome^®^ J405 mercury vapor analyzer) with a 0.01 µg m^−3^ resolution and a 750 ± 50 cc min^−1^ flow rate [53]. In the case of passive samplers, gold nanostructures have been used, for instance, by deploying very thin gold electrodes (50 nm) to Hg^0^ for 100 min, and then measuring the changes in resistance (Limit of Detection, LOD: 1 μg/m^3^): in this case, the analysis was provided by the same device [54]. Mercury also induces changes in the optical properties of gold films [55]. Exploiting this feature, in literature, porous glass discs were coated with AuNPs, showing a linear range within 0.1–15 ng Hg^0^ and a LOD of 0.4 ng [56]: the exposure to Hg^0^ caused a color change from red to violet-purple.

Here, the AuNP–TiO_2_NP layer was exposed to a known concentration of elemental mercury vapors (14 mg/m^3^) at increasing time (room temperature, 35% RH).

**Figure 4:**
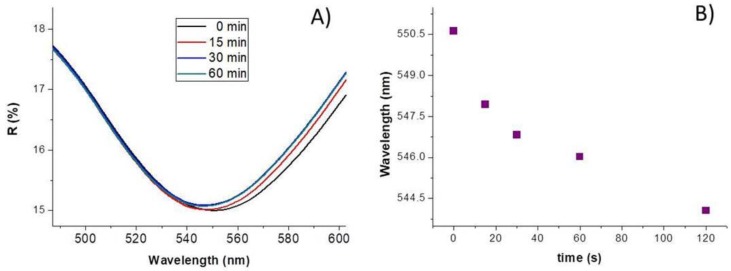
Comparison of UV-Vis diffuse reflectance spectra of AuNPs/TiO_2_ layer in a 450–650 nm wavelength range at different exposure times to Hg^0^ vapor pressure at 20 °C and 35%RH (**A**); a curve plot depicting the wavelength values of the AuNPs/TiO_2_ layer at increasing exposure time to Hg^0^ (**B**).

The diffuse reflectance spectra (Figure 4A) depicted an apparent wavelength blue shift of ≈ 2.7 nm after 15 min of exposure, up to ≈ 4.6 nm, and ≈ 6.6 nm after 60 and 120 min, respectively, according to a non-linear curve (Figure 4B), suggesting a quicker process initially and slower afterwards, also reported in literature [57]. A possible explanation has been provided by Mie theory [58]: since mercury is expected to be adsorbed strongly onto gold surface and Au is more electronegative than Hg (2.5 and 2.0, respectively) [59], mercury may be able to donate the electron density to gold NPs [60] causing the surface plasmon mode to blue-shift [58]. The related changes (0.1%) in reflectance, which seem to increase with dependence on the exposure time (up to 60 min), may be due to the change of the refractive index for mercury entrapment inside the nanocomposite layer [49]. 

**Figure 5:**
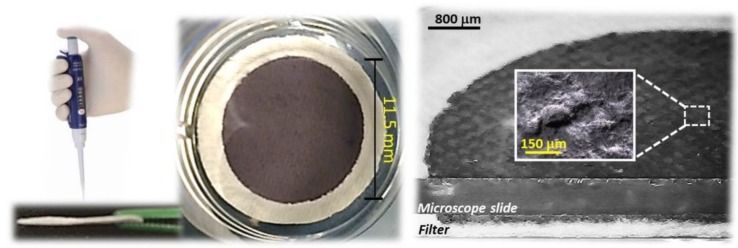
(**Left**) Drop deposition of TiO_2_/AuNPs aqueous suspension; top view (**middle**) and side view (**right**) of the resulting disc. The inset depicts an optical magnification of the film surface.

Similarly, a coating dispersed on a fibrous quartz surface of about 0.99 × 10^2^ mm^2^ was achieved by slowly dropping hundreds microliters of the aqueous suspension through a suitable mask, defining the area to be covered, and allowing water in excess to flow away through the disk filter. After deposition, the layer looked quite compact and it conformed to the quartz disc surface (Figure 5). The coating penetrated inside the quartz filter for about 25% of its thick layer, confirmed by the color change of cross filter section in Figure 5, right. Optical micrographs show that the composite film was conformal to the microfibrous surface of the supporting scaffold (Figure 5, inset), keeping its roughness characterized by valleys and overhangs.

**Figure 6:**
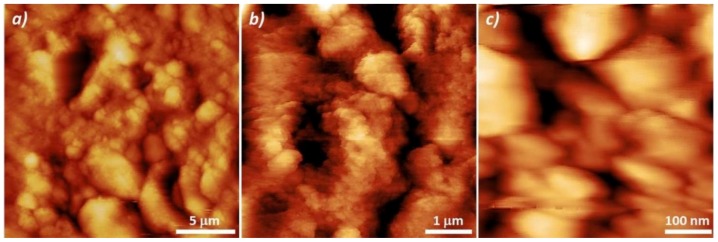
Atomic Force Microscope topography images of the quartz filter surface coated with a TiO_2_/AuNP layer, at different magnifications.

Such a layer, since partially withheld by quartz microfibers, was stable and easy to be handled without suffering apparent damages or detachment. Figure 6 presents Atomic Force Microscope (AFM) surface topography images of the functionalized quartz filters. The coating surface showed a rough material provided of different sized grains (Ra: 5.4 ± 1.4 nm, average roughness) with ridges and valleys conforming to the fibrous substrate (Figure 6a). At higher magnification (Figure 6b,c), grains (Ra: 140 ± 85 nm) appeared densely packed with a series of void spaces (darker areas), due to their uneven boundaries.

**Figure 7:**
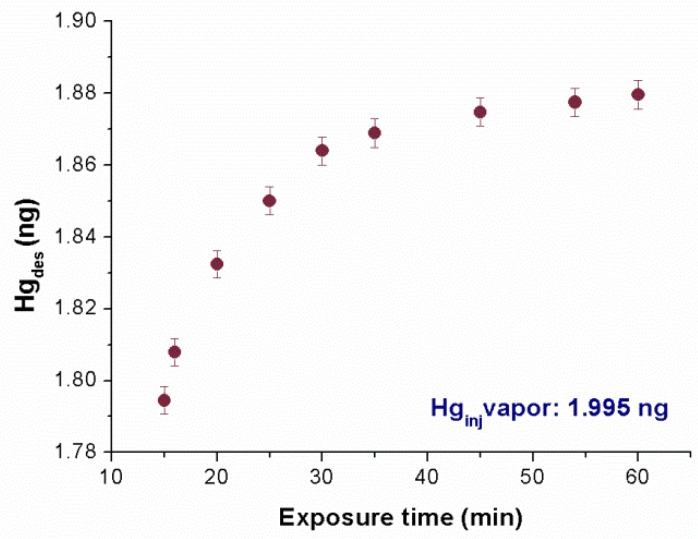
Adsorption curve of a known amount of injected Hg^0^ vapors (C: 0.235 ng/mL) versus exposure times ranging between 15 and 60 min.

In order to calibrate the adsorbing membrane to mercury vapors, the adsorbing disc was placed on the bottom of a suitable sealed glass chamber that was 2.5 cm height, and with a volume of 8.5 mL, where increasing amounts of Hg^0^ vapors (µL) were injected by a gas-tight syringe at ~20 °C (under dry air). The injected volumes were selected to lower the experimental error as much as possible. Further, each amount was first theoretically estimated and then experimentally measured by injection into the analytical instrument. Finally, the disc was subjected to thermal desorption under an air flow that collected the desorbed mercury and delivered it to the mercury analyzer. In Figure 7, the adsorbed mass of Hg^0^ versus the exposure time is reported. It was noted that 15 min of film exposure appeared to be sufficient to adsorb 90% of the injected mass. However, all of the estimated injected mass values were not completely adsorbed, even after an hour, probably due to the partial nonspecific sorption of mercury to the glass container.

**Figure 8:**
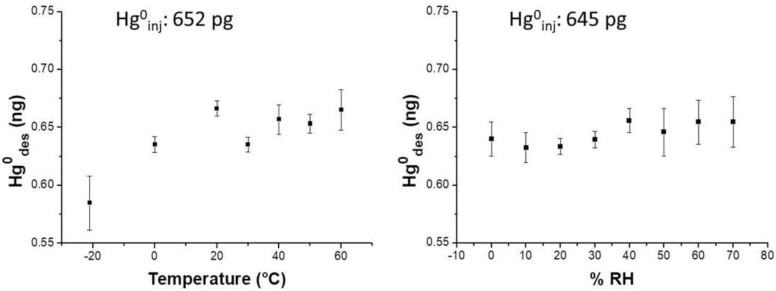
Plots depicting the desorbed mercury from a AuNP/TiO_2_NP film upon exposure, within a sealed glass vessel that is similar to the passive sampler (PAS) container, to: (**left**) a known mass of Hg^0^ vapor (about 652 ± 0.01 pg) when temperature of the sampler changed (ranging between −20 and 60 °C; dry air); (**right**) a known mass of Hg^0^ vapor (about 645 ± 0.01 pg) when the %RH in the sampler changed (ranging between 5% and 70% relative humidity; room temperature). Each point in both the graphs is the resulting mean value from 5-times repeated-measures (error bars depict standard deviations).

Therefore, to investigate the effects of humidity and temperature, further measurements of adsorption at different Hg^0^ vapor concentrations were carried out by exposing the same membrane to the pollutant just for 15 min. The relative humidity changes were controlled and generated by a mass flow controller flowing dry air, and increasing the concentrations of the water vapors throughout the measuring chamber. Temperature values were provided by dipping the measuring chamber into a thermal bath. In order to obtain the membrane performances at −20 °C, the measuring chamber was put into a fridge at −20 °C. After exposure to Hg^0^ by injection (15 min) the sample was resumed and desorbed for quantification. Experimental results reported a slight increase of the adsorbed mass when the relative humidity increased up to 70% RH (relative humidity) within the measurement chamber. Specifically, when 645 pg of Hg vapor were injected into the differently humidified measuring chamber, the nanostructured material was slightly affected by the water molecules, improving entrapment by an additional amount of 0.4 ± 0.01 pg of the analyte per %RH unit (Figure 8, right). 

Notably, when %RH was ranging between 50–70%, the desorbed values oscillated between 0.632 ng and 0.656 ng, i.e. they increased the values spread, and then the error. Similarly, temperature also slightly affected the analyte adsorption onto the PAS membrane, since the curve slope increased by 0.62 pg per Celsius degree over a thermal range of −20–60 °C when approximately 645 pg Hg^0^ mass was injected (Figure 8, left).

**Figure 9:**
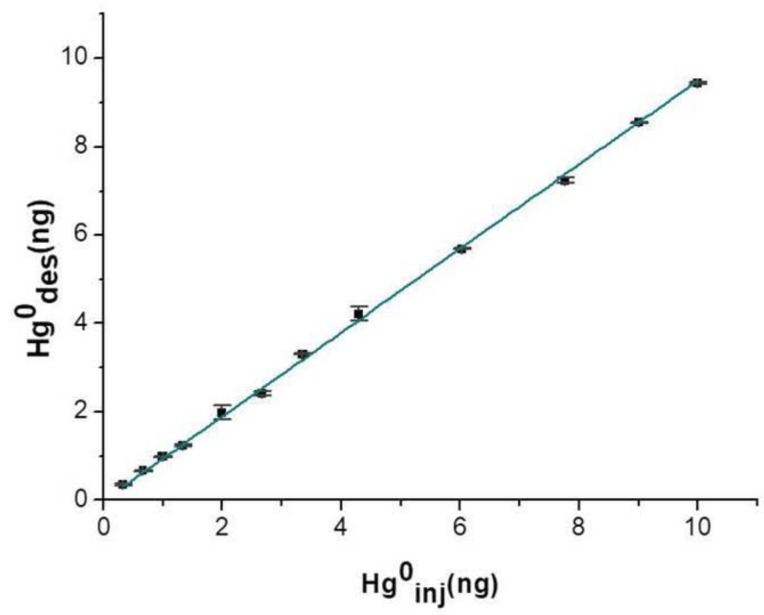
Adsorption curve of mercury vapors (measured as PAS-desorbed mercury) at increasing injected amounts of mercury vapors (ng).

Injected mercury vapor mass values were compared to the amount of Hg^0^ that was actually adsorbed onto the exposed layer, in order to value its efficiency. Such a parameter was measured by adding a few microliters containing increasing masses of Hg^0^ vapor into the measuring chamber at room temperature and in dry air. Five replicate measurements were provided for each injection. Upon a 15 min-deployment time, the adsorbing disc was thermally desorbed, and the collected vapors were delivered to the analytical instrument and then measured. The linear relationships between the mass injected and the mass adsorbed until 10 ng were depicted in the plot of Figure 9, suggesting a high absorbance of the nanostructured material. The affinity between mercury and the nanostructured material was confirmed also by the slope value of the linear fitting (*S*: 0.950 ± 0.005, *R*^2^: 0.998) calculated on 15 min of sampling per each step. The mercury adsorbed mass when the disc was exposed to saturated mercury vapors for 18 h (*T:* 20 °C) was estimated to be more than 15 μg, confirming that such a thin layer was a very highly sorbent device for mercury.

**Figure 10:**
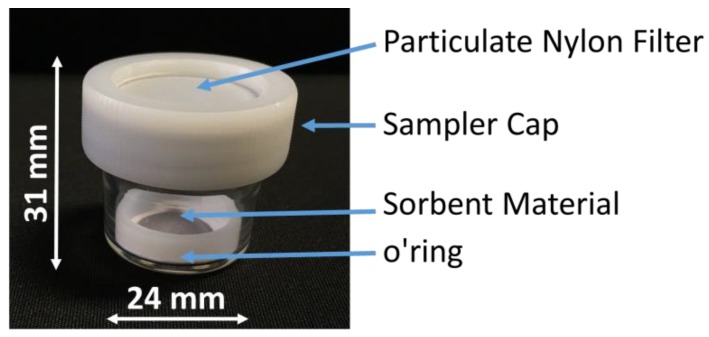
Prototype of mercury passive sampler comprising a diffusive membrane (particulate filter), the fibrous disc coated with AuNP/TiO_2_NP (sorbent material), and a borosilicate vessel allowing the axial diffusion of Hg^0^ from the cap to the bottom. The O-ring stops the disk on the tube bottom.

Samples were completely restored after dozens of cycles of measurements, confirming the potential to use the same sample for many exposures. The functioning of the diffusive samplers is based on the movement of the contaminant molecules across a concentration gradient. In the collecting device (the case of a passive sampler, Figure 10) the contaminants diffuse from an area of higher concentration towards an area of lower concentration. According to the first Fick’s Law, the rate at which the chemicals diffuse is represented by the following formula:

(1) Q=D(AL)C t,

where *Q* is the amount of the sample collected (ng), *D* is the diffusion coefficient (cm^2^/min), *A* is the cross-sectional area of the diffusion path (cm^2^), *L* is the diffusive path length (cm), *C* is the airborne concentration (mg/m^3^) and *t* is the sampling time (min). Each contaminant has its own diffusion coefficient that is determined by its unique chemical and physical properties. The *A/L* parameter is determined by the sampler’s geometry; the product of *D (A/L)* is the theoretical sampling rate of a diffusive sampler for a specific compound (e.g., elementary mercury).

**Figure 11:**
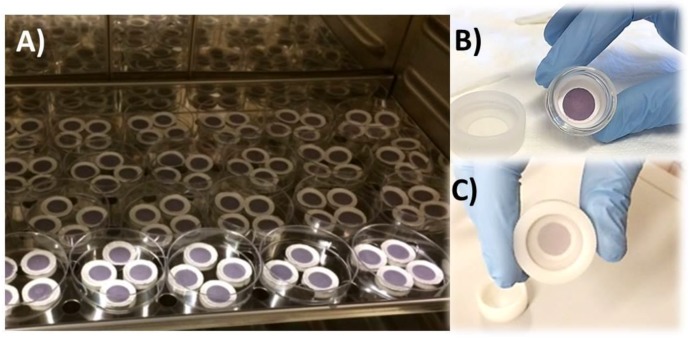
A fabrication step of the PAS devices: many of AuNPs-TiO_2_/quartz discs drop-deposited and placed in an oven to facilitate the solvent evaporation (**A**); the placement of the adsorbing disc on the borosilicate vessel bottom (**B**); the PAS device with the particulate filter mounted (**C**).

In order to experimentally evaluate the sampling rate (*SR*) of the proposed passive sampler, a useful method is given by the use of the empirical equation (2) [28]:

(2) SR=Q/(Ct)

where *Q* is the amount of the adsorbed mercury (ng), *C* is the exposition concentration (ng m^−3^), and *t* is the deployed time (days) of the sample, named as PS. In our case, several measurements have been performed, exposing three passive samplers (PS1, PS2, PS3) at three different vapor mercury concentrations for 3, 7, and 15 days of sampling time. All of the measurements were performed in three measuring chambers, where three different ambient vapor mercury concentrations, of 1.2, 3.5, and 4.5 ng m^−3^, were kept constant. As previously mentioned, the PAS device here described works exploiting the unassisted axial diffusion process of the mercury vapor through the diffusive membrane, along the glass vessel (diffusion path), up to the adsorbing film placed on the vessel bottom. This PAS comprises a see-through borosilicate vessel, a cap made of a nylon membrane for gas diffusion and particulate stopping, a locking ring to keep the adsorbing membrane to the vessel bottom, and finally the adsorbing membrane (the violet discs in Figure 10 and Figure 11). The PAS fabrication was easy and quite reproducible, since all of the membranes that were decorated by a given volume of the suspension, reported the same weight (10.00 ± 0.25 mg), obviously with the uncertainty (2.5%) generated by the deposition technique and by the irregularity of the hosting substrate. The resulting nanostructures looked very stable, since they were partially entrapped inside the filter and did not appear to be decolored or scratched, even after 1 year of measurements. The pictures in Figure 11 shows a batch of the fibrous quartz discs decorated with the AuNP–TiO_2_NP layers, and their placement into the device in order to be characterized as potential PAS for mercury. Each adsorbing disc was mercury thermally desorbed before (to have a clean substrate) and after (to measure the concentration in air) each exposure, and the desorbed vapors were delivered to the analytical instrument. Commonly, 10 min of heating was sufficient to both restore the adsorbing disc, and to measure the amount of Hg^0^ that was adsorbed throughout the deployment time. For each measure, quartz discs were heated under clean and dry air flow until the Tekran Analyzer displayed values of Hg^0^ that were close to zero. 

After each exposure, using Equation (2), the sampling rate (*SR*) of each passive was calculated, and the relative results were reported in Table 1.

**Figure 12:**
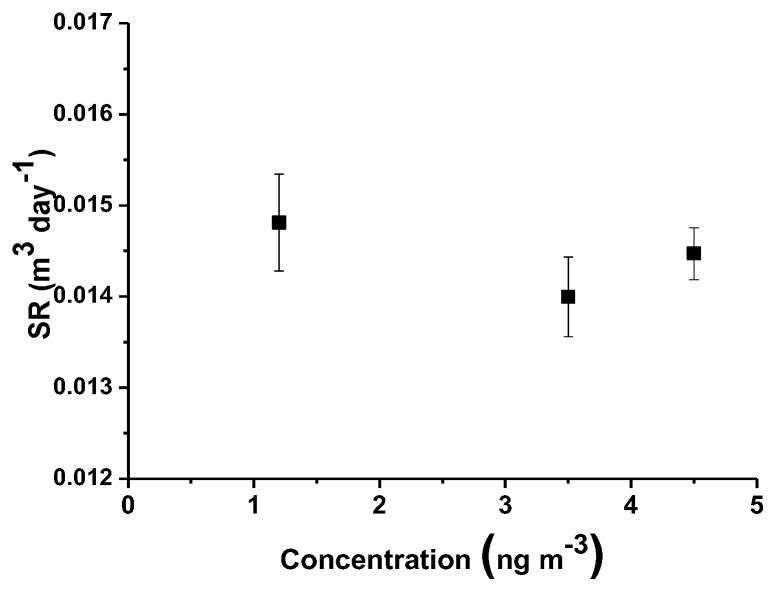
Estimated sampling rate vs concentration (ranging from 1.2 to 4.5 ngm^−3^).

The mean value of sampling rate was estimated to be 0.014 ± 0.0007 (m^3^/day). Figure 12 depicts the *SR* values related to the exposure to increasing concentrations of mercury vapors. The reported error bars are referred to the standard deviation (*SD*) of uncertainty, showing a low dispersion of the overall values at increasing concentrations. In literature, generally depending on the range of environmental mercury concentration to be monitored, several PASs have been designed with different *SRs* [28], ranging from 0.00031 m^3^/day based on gold-coated silica placed in an axial tube without diffusive membrane [61], to 0.13 m^3^/day based on sulfur-impregnated activated carbon (axial tube, no diffusive membrane) [38,42], and to 6.6 m^3^/day based on gold-coated quartz fiber filters [62]. Furthermore, since *SRs* are commonly affected by environmental conditions (strong wind, pressure, proximity to the coast or to desert and sandy areas, rain), protective shells have also been also used, improving the reliability of the measurements (*SR*: 0.121 ± 0.005 m^3^/day) [63]. Physically, *SR* quantifies the volume of air that is effectively stripped of the pollutant per unit of time. As previously described, it depends on the diffusion coefficient of the compound in air, but also on other parameters as the diffusive path length of the PAS device: by changing parameters as the length or the quality of the diffusive barriers, *SR* can be modulated. Thus, higher *SR* values are commonly preferred when very low-polluted environments may be measured with a certain accuracy and for a short time (e.g., wearable devices that are commonly suitable for 1 or 2 deployment days). Conversely, PASs with lower *SR* values are desired for longer times of monitoring (up to one year) to ensure the presence of free adsorbing sites on the membrane. On the other hand, *SR* values that are too low could be responsible for a low resolution of the PAS devices when exposed in poorly polluted sites, making them unattractive as accurate measurement tools, since, accordingly, highly sensitive analytical techniques are required.

**Figure 13:**
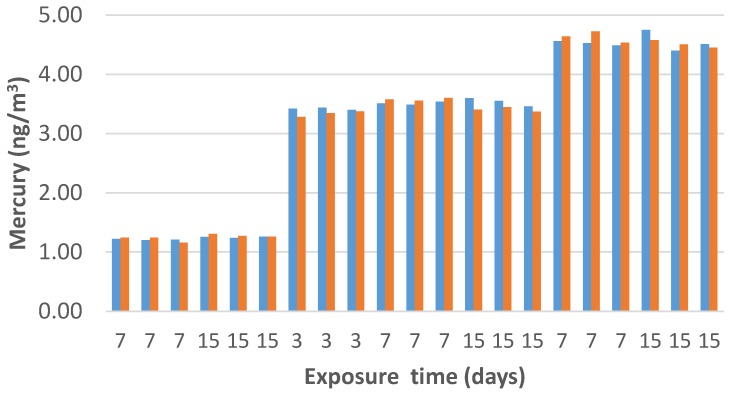
Comparison between the PAS estimated concentration using the experimental sampling rate (red bar) and the measured values by the vapor mercury analyzer (blue bar).

A comparison between the estimated concentrations, calculated using the experimental sampling rate (*SR*) and the measured values by the vapor mercury analyzer, has been reported in Figure 13.

The resulting PAS values were similar to those that were reported by the analytical device over exposure times ranging between three and 15 days and to different concentrations of Hg^0^ vapors, ranging between 1.2 and 4.6 ng/m^3^. Specifically, when the mercury analyzer measured average concentration values of 1.23, 3.49, and 4.59 ng/m^3^, PAS values were reported to be 1.25, 3.44, and 4.57 ng/m^3^, respectively, with an average deviation of ~1.2%.

## 4. Conclusions

Since thin film structures are preferred for the development of passive samplers, a nanostructured mercury vapor-adsorbing layer made of TiO_2_NPs and photo-decorated with AuNPs was designed and assembled by drop-casting onto a microporous filter of SiO_2_. The decorated disc looked quite compact and it conformed to the SiO_2_ microfibrous surface, keeping its roughness made of valleys and overhangs, and penetrating inside the quartz filter for less than a quarter of its thickness. Such a resulting layer was stable and easy to be handled without suffering apparent damage or detachment.

The gold nanoparticles, grown on TiO_2_NPs, shared similar size with the support grains, assembling together into homogeneous aggregates. However if the AuNP shapes were regular (spherical, highly crystalline), their size distribution became heterogeneous, ranging between 5 and 40 nm. 

Exploiting both the high surface/volume ratio and the strong affinity between gold and mercury, the membranes, investigated as potential passive samplers for gaseous elemental mercury, showed a high absorbency (up to 15 μg), together with a 95% efficiency of absorption, with only slight effects due to temperature (+0.1% per Celsius degree, in a thermal range between −20 and 60 °C) and relative humidity (+0.06% per %RH unit, between a dry and a 70% humid environment). 

Samples could be restored after dozens of cycles of measurements by thermal desorption, confirming their potential to use the same sample for many exposures.

When the adsorbing discs were placed inside axial passive samplers, a sampling rate of 0.014 m^3^/day was estimated when they were tested in controlled environments. Their features were compared to those of the analytical measuring instrument, reporting an average deviation of ~1.2%. Such a value suggested the chance to be applied for both short and longer monitoring campaigns. Therefore, due to their ease of preparation, their high sensitivity to gaseous elemental mercury due to the strong affinity between mercury and gold, high efficiency and a long lifetime, the AuNP/TiO_2_NP-based devices are expected to be promising candidates for passive sampling strategies.

On the other hand, further studies are needed to evaluate the effect of thickness on the efficiency and adhesiveness of the quartz support. Similarly, the AuNP size and shape, as well as its density will be investigated too, in order to assess their effects onto the sensitivity and compactness of the aggregated hybrid nanoparticles inside the film. Finally, further investigations are in progress in environments that are polluted with potential interferents, such as chlorides and sulfides, and in extreme environmental conditions, in order to be considered for monitoring campaigns over the globe.

## Figures and Tables

**Table 1:** Passive devices sampling rate values.

Sampler	^(1)^Hg^0^ Vapor Concentration (ng/m^3^)	Hg^0^ Adsorbed Mass (ng)	Exposure Time (days)	Sampling Rate (m^3^/days)
PS1	3.5	0.140	3	0.013
PS2	3.5	0.143	3	0.014
PS3	3.5	0.144	3	0.014
PS1	1.2	0.124	7	0.015
PS2	1.2	0.124	7	0.015
PS3	1.2	0.116	7	0.014
PS1	3.5	0.357	7	0.015
PS2	3.5	0.355	7	0.014
PS3	3.5	0.359	7	0.015
PS1	4.5	0.463	7	0.015
PS2	4.5	0.471	7	0.015
PS3	4.5	0.452	7	0.014
PS1	1.2	0.279	15	0.016
PS2	1.2	0.272	15	0.015
PS3	1.2	0.269	15	0.015
PS1	3.5	0.727	15	0.014
PS2	3.5	0.736	15	0.014
PS3	3.5	0.720	15	0.014
PS1	4.5	0.978	15	0.014
PS2	4.5	0.962	15	0.014
PS3	4.5	0.951	15	0.014

^1^ measured by TEKRAN 2537A unit, the temperature was 24 °C (with a fluctuation of 1 °C), and the RH% was 40% (with a fluctuation of 2%) over the whole experiment.
